# Reactive microglia and IL1β/IL-1R1-signaling mediate neuroprotection in excitotoxin-damaged mouse retina

**DOI:** 10.1186/s12974-019-1505-5

**Published:** 2019-06-06

**Authors:** Levi Todd, Isabella Palazzo, Lilianna Suarez, Xiaoyu Liu, Leo Volkov, Thanh V. Hoang, Warren A. Campbell, Seth Blackshaw, Ning Quan, Andy J. Fischer

**Affiliations:** 1 Department of Neuroscience, College of Medicine, The Ohio State University, 3020 Graves Hall, 333 W. 10th Ave, Columbus, OH 43210-1239 USA; 2 Department of Pathology and Immunology, Washington University School of Medicine, St. Louis, MO USA; 3 Solomon H. Snyder Department of Neuroscience, Johns Hopkins University School of Medicine, Baltimore, MD USA; 4 Institute for Behavioral Medicine Research, College of Medicine, The Ohio State University, Columbus, OH USA; 5 Division of Biosciences, College of Dentistry, The Ohio State University, Columbus, OH USA; 6 Department of Biological Structure, University of Washington, Seattle, WA USA

**Keywords:** Microglia, IL1β, IL-1R1, Retinal neuroprotection

## Abstract

**Background:**

Microglia and inflammation have context-specific impacts upon neuronal survival in different models of central nervous system (CNS) disease. Herein, we investigate how inflammatory mediators, including microglia, interleukin 1 beta (IL1β), and signaling through interleukin 1 receptor type 1 (IL-1R1), influence the survival of retinal neurons in response to excitotoxic damage.

**Methods:**

Excitotoxic retinal damage was induced via intraocular injections of NMDA. Microglial phenotype and neuronal survival were assessed by immunohistochemistry. Single-cell RNA sequencing was performed to obtain transcriptomic profiles. Microglia were ablated by using clodronate liposome or PLX5622. Retinas were treated with IL1β prior to NMDA damage and cell death was assessed in wild type, IL-1R1 null mice, and mice expressing IL-1R1 only in astrocytes.

**Results:**

NMDA-induced damage included neuronal cell death, microglial reactivity, upregulation of pro-inflammatory cytokines, and genes associated with IL1β-signaling in different types of retinal neurons and glia. Expression of the IL1β receptor, IL-1R1, was evident in astrocytes, endothelial cells, some Müller glia, and OFF bipolar cells. Ablation of microglia with clodronate liposomes or Csf1r antagonist (PLX5622) resulted in elevated cell death and diminished neuronal survival in excitotoxin-damaged retinas. Exogenous IL1β stimulated the proliferation and reactivity of microglia in the absence of damage, reduced numbers of dying cells in damaged retinas, and increased neuronal survival following an insult. IL1β failed to provide neuroprotection in the IL-1R1-null retina, but IL1β-mediated neuroprotection was rescued when expression of IL-1R1 was restored in astrocytes.

**Conclusions:**

We conclude that reactive microglia provide protection to retinal neurons, since the absence of microglia is detrimental to survival. We propose that, at least in part, the survival-influencing effects of microglia may be mediated by IL1β, IL-1R1, and interactions of microglia and other macroglia.

**Electronic supplementary material:**

The online version of this article (10.1186/s12974-019-1505-5) contains supplementary material, which is available to authorized users.

## Background

Microglia in the central nervous system have a significant impact upon neuronal function and survival following injury, and this impact can be beneficial or detrimental depending on the context [1]. Microglia are the innate immune cells of the central nervous system and are derived from erythro-myeloid precursor cells that originate in the yolk sac during early stages of development [2]. Prior to the onset of neurogenesis, microglia migrate into the retina through the vitreous and retinal periphery [3]. In the nervous system, microglia influence blood-vessel development, programmed cell death, phagocytosis of debris, and activity-dependent synaptic pruning [4, 5]. In response to injury, microglia rapidly migrate to the site of injury and undergo morphological and molecular changes associated with inflammation [1, 6]. Macrophages, another innate immune cell type, are often characterized by phenotypes that range across a spectrum; the “M1” activation profile is pro-inflammatory and cytotoxic, whereas the “M2” activation can promote tissue repair (reviewed by [7]). Microglia can also be activated in helpful and harmful manners (reviewed by [1]), although the existence of a simple polarized phenotype is doubtful [8, 9].

Upregulation of pro-inflammatory or neurotoxic molecules by activated microglia is widely considered to be deleterious to neuronal survival [10, 11]. Multiple lines of evidence support this notion in diseased or damaged retinas. Activated microglia phagocytize healthy rod photoreceptors in a mouse model of retinitis pigmentosa and this exacerbates degeneration [12]. In a mouse model of glaucoma, upregulation of complement occurs prior to the onset of ganglion cell death, which tags synapses of ganglion cells to be targeted for engulfment by microglia [13]. In support of this finding, inhibition of complement suppresses the degeneration of RGCs in glaucomatous mouse retinas [14]. In a mouse model of hemorrhagic macular degeneration, treatment with minocycline prevents microglia accumulation in the sub-retinal space and increased photoreceptor survival [15]. However, in the context of retinal detachment, ablation of microglia prevented their accumulation in the sub-retinal space and this decreased survival of photoreceptors [16]. Multiple lines of evidence also support the notion that reactive microglia can be neuroprotective. In the chick retina, microglia ablation exacerbates NMDA-induced neuronal death [17]. Similarly, microglia are protective against excitotoxic damage in NMDA-treated hippocampal slice-cultures [18]. Ablation of microglia resulted in an increase in hippocampal neuron death in response to NMDA, and replenishment of microglia restored the resistance of hippocampal neurons to excitotoxicity [18]. In some paradigms, microglia can support neuronal survival during injury, but become detrimental to survival during the recovery phase [19]. Considered together, microglia can be beneficial or harmful to neuronal survival and the survival-influencing actions of microglia are context-specific.

In this study, we investigate whether microglia influence neuronal survival following an excitotoxic insult in the mouse retina. In addition, we investigate the involvement of IL1β and cell type-specific IL-1R1-receptor signaling. Collectively, our findings suggest that reactive microglia provide neuroprotection in excitotoxin-damaged retinas and this may be mediated by microglial production of IL1β, which acts, in part, via IL-1R1 in macroglia.

## Methods and materials

### Animals

The use of animals in these experiments was in accordance with the guidelines established by the National Institutes of Health and the Ohio State University. Mice were kept on a cycle of 12 h light, 12 h dark (lights on at 6:00 AM). C57BL/6J mice between the ages of P40-P100 were used for all experiments except when noted. Lines of mice included IL-1R1 reporter mice (IL-1R1GR/GR), IL-1R1 null mice (IL-1R1-r/r), and GFAPCre-IL-1R1r/r [20]. GFAPCre-IL-1R1r/r mice are made from GFAP>Cre x homozygous knock in stop-fl/fl-IL1R1-3HA-IRES-tdTomato line of mice that was crossed onto the IL-1R1-null background [20, 21].

### Preparation of clodronate liposomes

The preparation of clodronate liposomes was similar to previous descriptions [22, 23]. Fifty nanograms of cholesterol and 8 mg egg lecithin were dissolved in chloroform in a round-bottom flask. The solution was evaporated until a white liposome residue remained. One hundred and fifty-eight milligrams of dichloro-methylene diphosphonate (clodronate) in sterile PBS was added and rotated for 10 min. The liposomes were sealed under N_2_ at room temperature for 2 h. Clodronate encapsulation was facilitated by sonication for 3 min. The liposomes were centrifuged at 10,000*×g* for 15 min and re-suspended in 150 ml PBS. We are unable to determine the clodronate concentration due to the stochastic nature of the clodronate combining with the liposomes. We tittered doses to levels where > 70% of the microglia were ablated at 1 day after treatment.

### Oral administration of PLX5622

C57BL/6 mice were fed chow formulated with PLX5622 (1200 ppm; provided by Plexxikon). Control animals were fed control chow AIN-76A (provided by Plexxikon). Mice were fed ad libitum on PLX5622 or control diets for a minimum of 2 weeks before experiments, and this diet was continued through the duration of each experiment.

### Intraocular injections

Mice were anesthetized by using an isoflurane/oxygen non-rebreathing inhaler; 98% oxygen and 2% isoflurane. Injections were made into the vitreous chamber of the eye through the dorsal sclera. Injections are made by using a 20-μl Hamilton syringe with a disposable custom 31-gauge needle with a cutting tip. The volume of all injections was 2–3 μl. For all experiments, the right eyes of mice were injected with the “test” compound and the contra-lateral left eyes were injected with vehicle as a control. Compounds were injected in 2 μl sterile saline. Compounds used in these studies included *N*-methyl-d-aspartate (NMDA; 38.5 or 154 μg/dose) and IL1β (200 ng/dose; R&D systems).

### scRNA-seq

Retinas were acutely dissociated via papain digestion and mild trituration. Dissociated cells were loaded onto the 10X Chromium Controller using Chromium Single Cell 3′ v2 reagents. Sequencing libraries were prepared following the manufacturer’s instructions (10X Genomics), with 10 cycles used for cDNA amplification and 12 cycles for library amplification. The resulting sequencing libraries were sequenced with paired end reads, with Read 1 (26 base pairs) and Read 2 (98 base pairs), on anNextseq500 at the Genomics Resources Core Facility (High Throughput Center) at Johns Hopkins University. Raw sequence data was processed with Cell Ranger software (10X Genomics) to align sequences, de-multiplex, and annotated to ENSMBL databases; count reads; assess levels of expression; and construct gene-cell matrices. *t*-Distributed Stochastic Neighbor Embedding (tSNE) plots were generated and probed using Cell Ranger and Cell Browser software (10X Genomics). The tSNE plots were generated via aggregate cluster analysis of 9 separate cDNA libraries, including 2 replicates of control undamaged retinas and retinas at different times after NMDA-treatment. The identity of clustered cells was established using known cell type-specific markers. Violin/scatter plots were generated using Seurat [24, 25]. Identification of cell types clustered together in tSNE plots was established by using a candidate approach using well-known cell type-specific markers, as follows: ganglion cells (*Pou4f2, Thy1, Nefl*), amacrine cells (*Tfap2a, Pax6, Gad1*), bipolar cells (*Vsx2, Grm6, Grik1*), horizontal cells (*Calb1, Lhx1*), rod photoreceptors (*Rho, Nrl, Nr2e3*), cone photoreceptors (*Opn1mw, Arr3*), astrocytes (*Pax2, S100b*), Müller glia (*Vim, Slc3a2, Glul, Rlbp1*), pericytes (*Tagln, Acta2*), and endothelial cells (*Tie1, Cldn5*).

### Fixation, sectioning, and immunocytochemistry

Tissues were fixed, sectioned, and immunolabeled as described previously [26–28]. None of the observed labeling was due to non-specific labeling of secondary antibodies or auto-fluorescence because sections labeled with secondary antibodies alone were devoid of fluorescence. Primary antibodies used in this study are described in Table 1. Secondary antibodies included donkey-anti-goat-Alexa488/568, goat-anti-rabbit-Alexa488/568, and goat-anti-mouse-Alexa488/568/647 (Thermo Fisher Scientific) diluted to 1:1000 in PBS plus 0.2% Triton X-100.

**Table 1:** Antibodies, sources, and working dilutions

Antigen	Working dilution	Host	Clone or catalog number	Source
Calretinin	1:1000	Rabbit	CR 7697	Swant Inc.
Draq5	1:2000	n/a	62251	Thermo Scientific
F4/80	1:250	Mouse	MCA497GA	Bio-Rad Laboratories
Iba1	1:1000	Rabbit	019-19741	Wako Pure Chemical Industries
Ki67	1:300	Rabbit	DRM004	OriGene
Pax6	1:1000	Rabbit	50-103-0098	Biolegend
Tomato Lectin	1:1000	n/a	B-1175	Vector Laboratories
GFP	1:500	Chicken	Ab13970	Abcam
CD31	1:1000	Mouse	102502	Biolegend
RFP	1:500	Goat	Ab8181-200	Origene Technologies
S100b	1:300	Rabbit	Ab227914	Abcam

### Terminal deoxynucleotidyl transferase dUTP nick end labeling (TUNEL)

To identify dying cells that contained fragmented DNA, the TUNEL method was used. We used an In Situ Cell Death Kit (TMR red; Roche Applied Science), as per the manufacturer’s instructions.

### Photography, measurements, cell counts, and statistics

Photomicrographs were obtained using a Leica DM5000B microscope equipped with epifluorescence and Leica DC500 digital camera. Confocal images were obtained using a Leica SP8 imaging system at the Department of Neuroscience Imaging Facility at the Ohio State University. Images were optimized for color, brightness, and contrast, multiple channels over-laid, and figures constructed by using Adobe Photoshop. Cell counts were performed on representative images. Counts were consistently made from central regions of retina that were within a 0.7-mm radius of the posterior pole of the eye.

Where significance of difference was determined between two treatment groups accounting for inter-individual variability (means of treated-control values), we performed a two-tailed, paired *t* test. Where significance of difference was determined between two treatment groups, we performed a two-tailed, unpaired *t* test. Where significance of difference was determined across multiple groups, we performed a one-way ANOVA, followed by Tukey’s test for multiple comparisons.

## Results

### Microglia reactivity accompanies NMDA-induced cell death

**Fig. 1:**
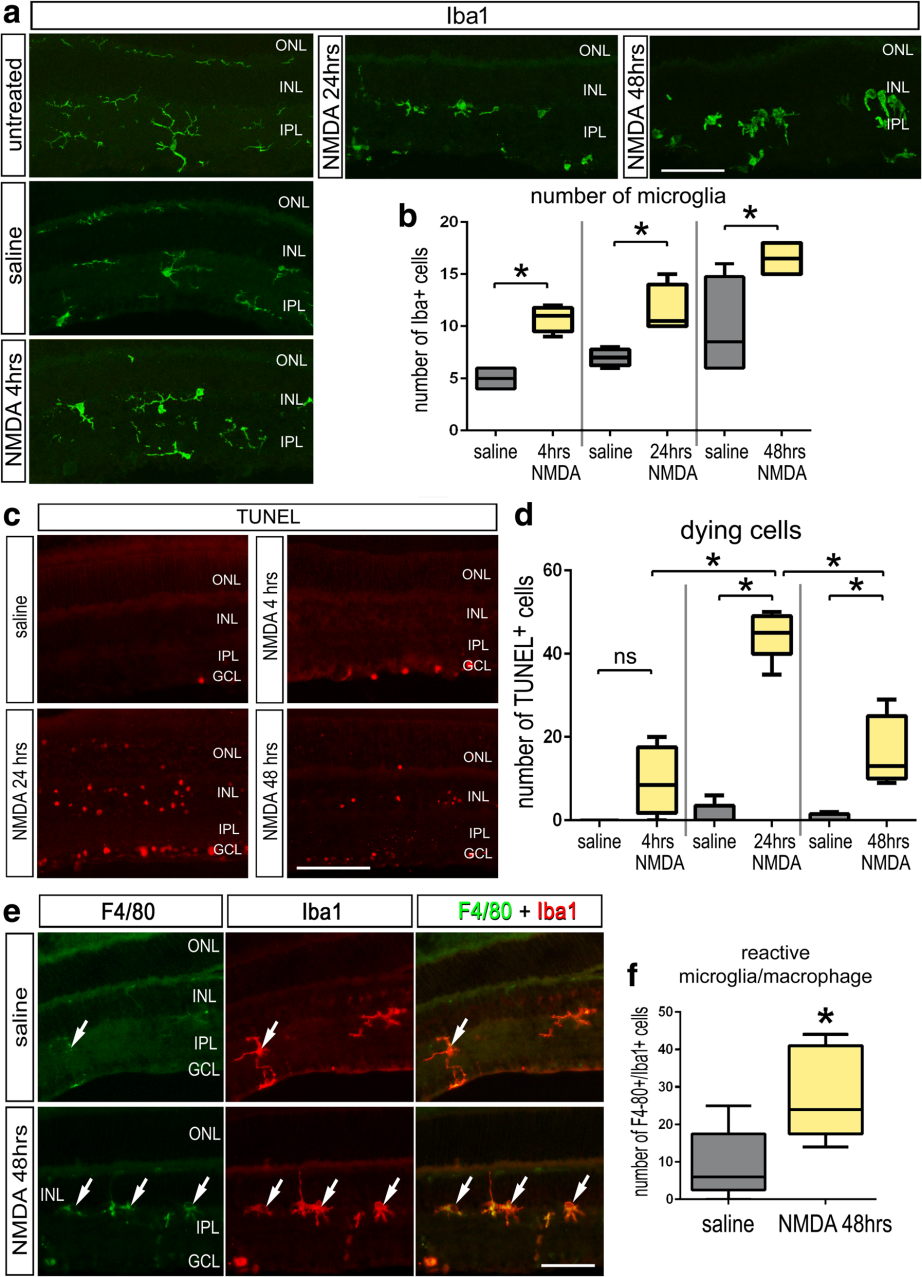
Reactive microglia accumulate in NMDA-damaged retinas. Eyes were injected with a single dose of NMDA and retinas harvested at different times after treatment. Sections were labeled with antibodies to Iba1 (**a**) and TUNEL (**c**), or antibodies to F4/80 (green) and Iba1 (red; **e**). The boxplots in **b** illustrate the mean, upper extreme, lower extreme, upper quartile, and lower quartile for total number of microglia in the retina at different times after NMDA-treatment (*n* = 4 animals).The boxplots in **d** illustrate the mean, upper extreme, lower extreme, upper quartile, and lower quartile for number of TUNEL-positive cells in the retina at different times after NMDA-treatment (*n* = 4 animals). The box plot in **f** illustrates the mean, upper extreme, lower extreme, upper quartile, and lower quartile for number of F4/80-positive/Iba1-positive cells (*n* ≥ 6 animals). Significance of difference (*p < 0.05) was determined by using a *t* test (**e**). Arrow-heads indicate microglia. The calibration bar panels **a**, **c**, and **e** represent 50 μm. Abbreviations: ONL, outer nuclear layer; INL, inner nuclear layer; IPL, inner plexiform layer; GCL, ganglion cell layer

Reactive microglia are known to be prevalent in damaged retinas [4]. We characterized the progression of reactive microglia and cell death in the retina following a single intravitreal injection of NMDA. Consistent with previous reports [29], we found that Iba1-postive microglia exhibited an amoeboid “reactive” morphology shortly after NMDA-damage. This morphology was prevalent among microglia at 24 and 48 h after treatment, and there was a significant increase in the total number of Iba1-positive microglia at 4 h, 24 h, and 48 h after NMDA (Fig. 1a, b). TUNEL-positive cells were detected as early as 4 h after NMDA-treatment (Fig. 1c, d). The abundance of dying cells peaked at 24 h and was diminished by 48 h after treatment (Fig. 1c, d). At 48 h after damage, we observed a significant increase in the number of Iba1-positive cells that expressed F4/80 (Fig. 1e, f); a marker that is upregulated by reactive microglia [30, 31]. TSPO has been reported as a marker for reactive microglia and has been implicated with the ligand Dbi as coordinating pro-inflammatory signals between microglia and Müller glia in the retina [32, 33]. However, we found that *TSPO* is upregulated in both microglia and Müller glia, whereas *Dbi* is upregulated in microglia and downregulated in Müller glia in NMDA-damaged retinas (Additional file 1: Figure S1), consistent with a recent report [34]. The antibodies to Iba1 and F4/80 do not distinguish between microglia and macrophage that may have migrated into the retina. Thus, when describing Iba1/F4/80-positive microglia, it is implied that these cells may include macrophages.

### The ablation of microglia exacerbates cell death in damaged retinas

**Fig. 2:**
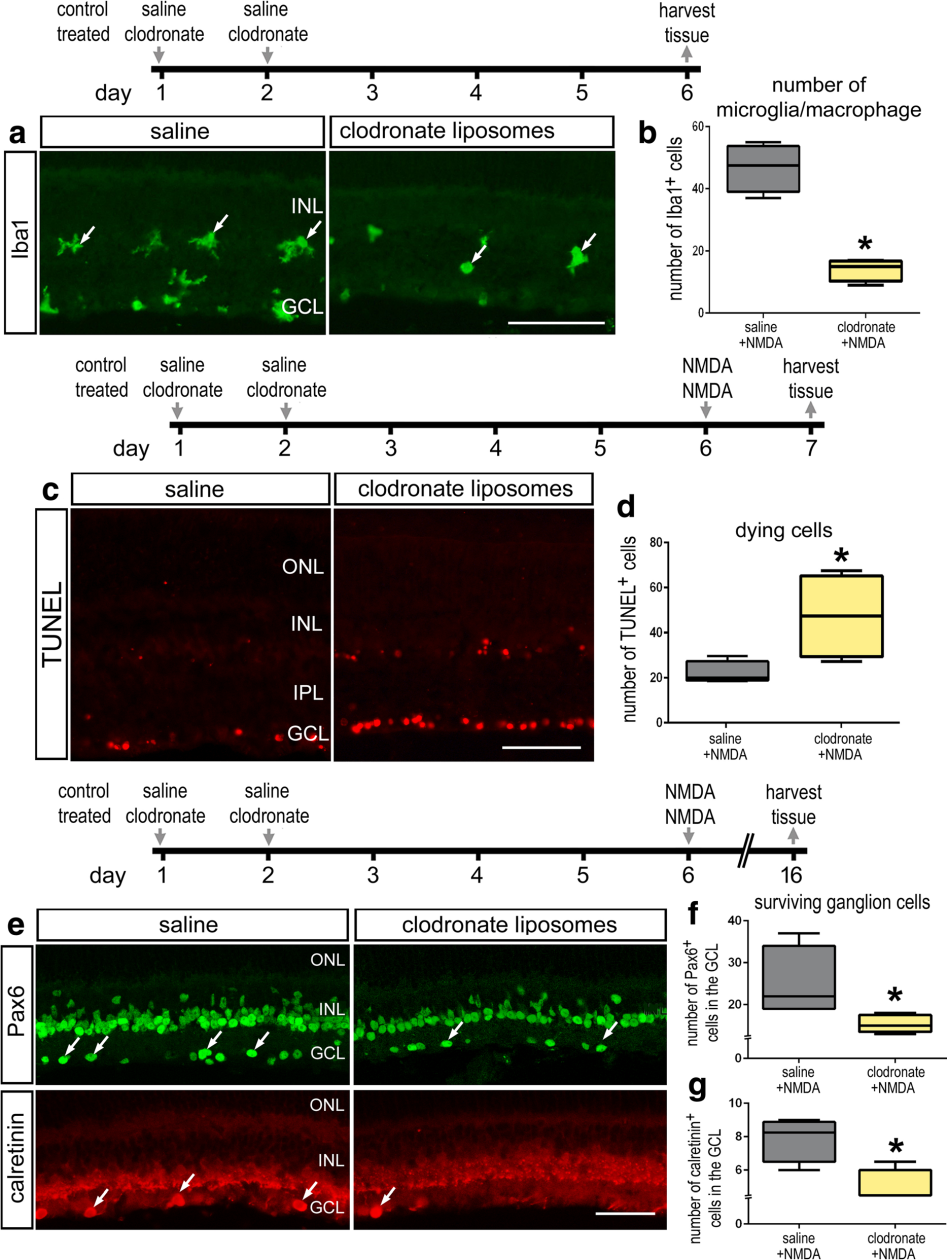
Clodronate-liposome-mediated ablation of microglia results in increased cell death and diminished neuronal survival in damaged retinas. Eyes were treated with 2 consecutive daily injections of saline or clodronate-liposomes and tissue harvested 4 days later (**a**, **b**), injected with NMDA at 4 days after clodronate-liposomes and harvested 1 day later (**c**, **d**), or injected with NMDA at 4 days after clodronate-liposomes and harvested 10 days later (**e**–**g**). Sections of the retina were labeled with antibodies to Iba1 (**a**), TUNEL (**c**), antibodies to Pax6 (green; **e**), or calretinin (red; **e**). The box plots in **b**, **d**, **f,** and **g** illustrate the mean, upper extreme, lower extreme, upper quartile, and lower quartile (*n* ≥ 6 animals). Significance of difference (*p < 0.05) was determined by using a *t* test. Arrows indicate microglia (**a**) or surviving ganglion cells (**e**). The calibration bars panels **a**, **c,** and **e** represent 50 μm. Abbreviations: ONL, outer nuclear layer; INL, inner nuclear layer; IPL, inner plexiform layer; GCL, ganglion cell layer

We investigated whether the ablation of microglia/macrophages in the retina influenced the survival of retinal neurons after an excitotoxic insult. Two consecutive daily intraocular injections of clodronate-liposomes depleted nearly 70% of the Iba1-positive microglia/macrophages in the retina (Fig. 2a, b). Empty liposomes were not used as a control because the liposomes potently stimulate the reactivity of microglia [22]. We did not detect any dying, TUNEL-positive cells in undamaged retinas following treatment with clodronate-liposome (not shown), suggesting that the clodronate-liposomes do not directly influence survival of retinal neurons or macroglia and that the destruction of the microglia is rapid and/or does not involve the fragmentation of DNA. With the majority of the microglia missing from the retina, we found more than a twofold increase in the number of TUNEL-positive cells in the retina after NMDA-treatment (Fig. 2c, d). The increase in cell death corresponded with decreased numbers of Pax6- and calretinin-positive cells in the ganglion cell layer (GCL) at 10 days after NMDA-treatment (Fig. 2e, f).

**Fig. 3:**
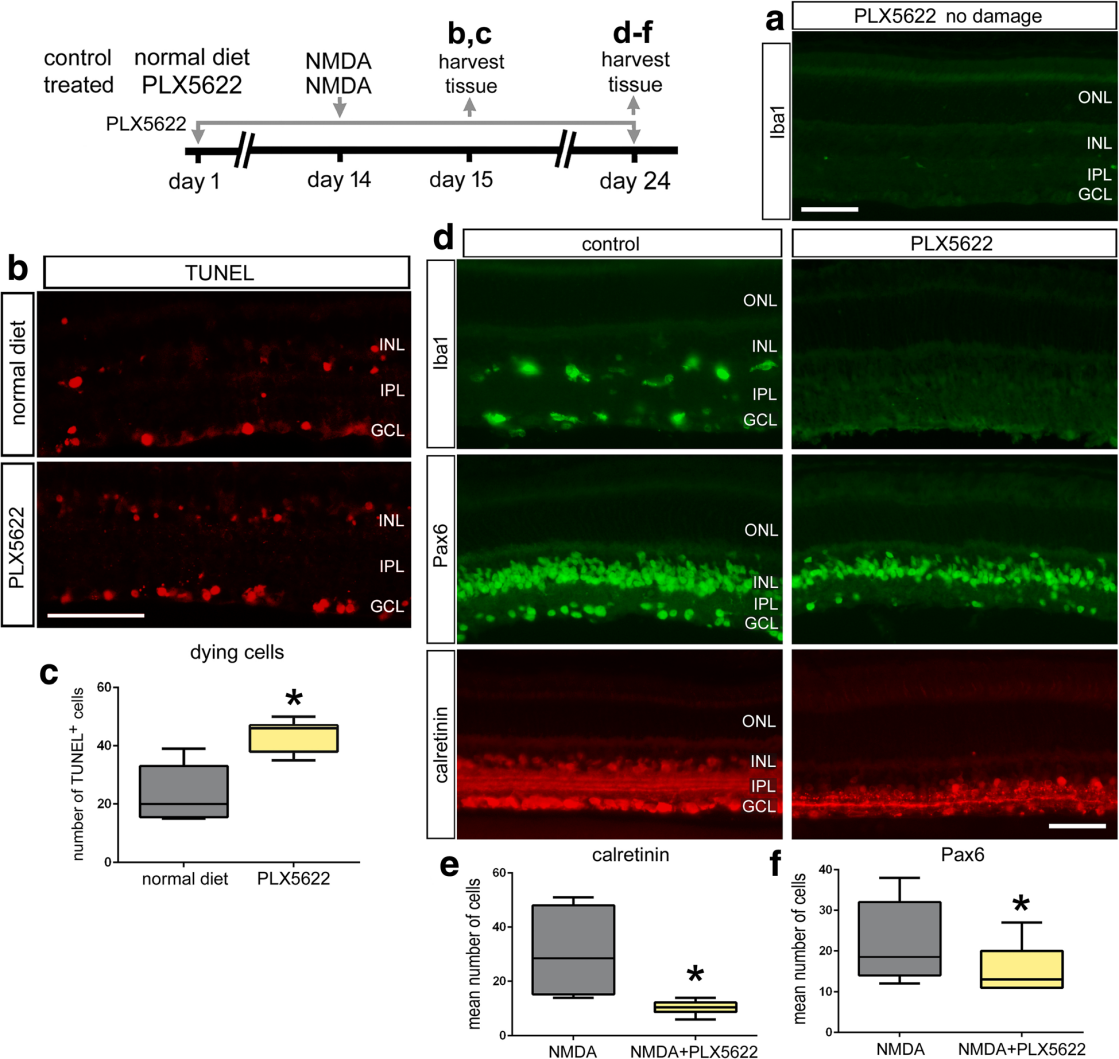
PLX5622-mediated ablation of microglia results in increased cell death and diminished neuronal survival in damaged retinas. Mice were fed control diet or diet that included PLX5622 for 2 weeks. After 2 weeks of treatment with PLX5622, eyes were injected with NMDA and retinas harvested at 1 (**b, c**) or 10 days (**d**–**f**) later. Retinal sections were labeled using the TUNEL method (**b**), antibodies to Iba1 (green; top) (**a, d**), Pax6 (green; middle) (**d**), or calretinin (red) (**d**).The box-plots **c**, **e**, and **f** illustrate the mean, upper extreme, lower extreme, upper quartile, and lower quartile (*n* ≥ 6 animals). Significance of difference (*p < 0.05) was determined by using a *t* test. The calibration bars panels **a**, **b,** and **d** represent 50 μm. Abbreviations: ONL, outer nuclear layer; INL, inner nuclear layer; IPL, inner plexiform layer; GCL, ganglion cell layer

It is possible that the few remaining microglia that evade destruction with the clodronate-liposomes exacerbate the neuronal death resulting from NMDA-treatment. Thus, as an alternative means of ablating retinal microglia, we treated mice with chronic exposure to Csf1r antagonist (PLX5622), which selectively ablates microglia in the CNS [35]. Consistent with previous reports [36], we found a near-complete ablation of microglia from the retina after 2 weeks of PLX5622-treatment (Fig. 3a). In retinas treated with PLX5622 and NMDA, we found that less than 1% of the Iba1-positive cells survive exposure to PLX5622 (Fig. 3d). With the ablation of nearly all microglia at the time of injury, we found more than a twofold increase in the number of TUNEL-positive cells in the inner nuclear layer (INL) and GCL after NMDA-damage (Fig. 3b, c). Consistent with our findings in clodronate-treated retinas, there was a decrease in numbers of Pax6- and calretinin-positive neurons at 10 days after NMDA-damage in PLX5622-treated retinas (Fig. 3d–f).

### Single-cell RNA-seq; expression of IL1β signaling components in the retina

**Fig. 4:**
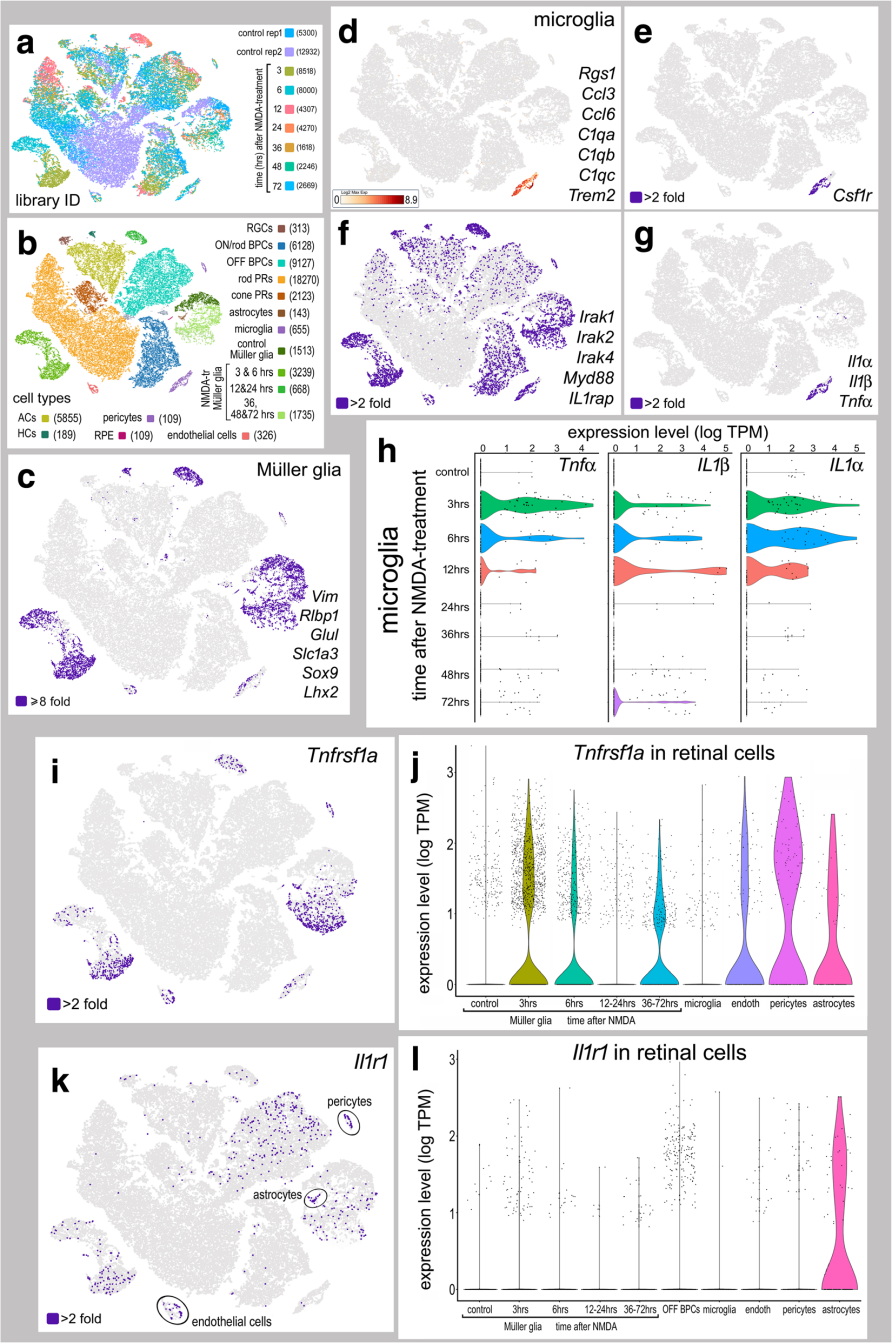
Expression of inflammatory cytokines and IL1-receptor-related genes in retinal cells following NMDA-treatment. scRNA-seq was used to identify patterns of expression of IL1β-related genes among acutely dissociated retinal cells. Each dot represents one cell. Cells were sampled from control retinas (rep1 5300 cells and rep2 12,932 cells), and from retinas at 3 h (8518 cells), 6 h (8000 cells), 12 h (4307 cells), 24 h (4270 cells), 36 h (1618 cells), 48 h (2246 cells), and 72 h (2269 cells) after NMDA-treatment (**a**). tSNE plots revealed distinct clustering of different types of retinal cells and numbers of cells surveyed (in parentheses) (**b**). Müller glia were identified based on significant (≥ 8-fold; purple dots) collective expression of *Lhx2*, *Sox9*, *Rlbp1,* or *Slc1a3* (**c**). Microglia were identified based on collective expression of *Rgs1, Trem2, Ccl3, Ccl4, C1qa, C1qb,* and *C1qc* (**d**). **e** t-SNE plot for expression (≥ 2-fold; purple dots) of *Csf1r,* the target of PLX5622, which was detected only in microglia. **f** t-SNE plot for the collective expression (≥ 2-fold; purple dots) of components of IL1-receptor signaling, *Irak1, Irak2, Irak4*, *IL1rap,* and *Myd88*. **g** t-SNE plot for the collective expression of *IL1α, IL1β, and TNFα*. Violin/scatter plots of *IL1α, IL1β, and TNFα* in microglia at different times after NMDA-treatment (**h**). **i** t-SNE plot for expression (≥ 2-fold) of *Tnfsfr1a* in endothelial cells, astrocytes, OFF bipolar cells, and Müller glia. **j** Violin/scatter plots of *Tnfsfr1a* in microglia, endothelial cells, pericytes, astrocytes, and Müller glia at different times after NMDA-treatment. **k** t-SNE plot for expression (≥ 2-fold) of *Il1r1* in endothelial cells, astrocytes, OFF bipolar cells, and Müller glia. **l** Violin/scatter plots of *Il1r1* in microglia, endothelial cells, pericytes, astrocytes, and Müller glia at different times after NMDA-treatment

To identify and provide a context for the IL-1-related factors that may mediate signaling in the different retinal cell types, we performed single-cell RNA sequencing (scRNA-seq) on NMDA-damaged mouse retina (Fig. 4). tSNE plots were generated from aggregate sequence data, 9 different single-cell cDNA libraries, including two preparations (rep1 and rep2) from undamaged retinas, and cells from retinas at 3, 6, 12, 24, 36, 48, and 72 h after NMDA-treatment (Fig. 4a). tSNE plots revealed distinct clustering of different retinal cell-types (Fig. 4a, b). The Müller glia were identified based on distinct and elevated expression, compared to all other cell types, of *Vim*, *Lhx2*, *Sox9*, *Rlbp1,* and *Slc1a3* (Fig. 4c). Relatively few astrocytes (143 cells) were identified, based on expression of *S100β* and *Pax2*, (not shown) and were clustered in close proximity to the Müller glia. The small number of astrocytes reflects the relatively low abundance of astrocytes among retinal cell types. However, we cannot exclude the possibility of capture-bias wherein cell dissociation methodologies and micro-fluidic capture impacts the chances of capturing particular cell types. The clusters of bipolar cells were identified based on combined expression of *Vsx2*, *Otx2*, *Lhx3, Grm6,* and *Grik1* (not shown). The cluster of endothelial cells was identified based on combined, distinct expression of *Tie1* and *Cldn5* (not shown). Microglia were identified based on combined, distinct expression of *Rgs1, Ccl3, Ccl6, C1qa, C1qb, C1qc, Trem2,* and *Csf1r* (Fig. 4d, e). The pattern of expression of *Csf1r* is consistent with the specific actions of PLX5622 at this receptor and selective ablation of microglia; *Csf1r* was not detected in significant numbers in retinal neurons, astrocytes, endothelial cells, or retinal pigmented epithelial (RPE) cells (Fig. 4e), suggesting that the diminished neuronal survival observed in PLX5622-treated animals resulted from specific actions at *Csf1r* expressed by microglia in the retina. Very few microglia from control, undamaged retinas were surveyed by scRNA-seq; less than 5% of the total number of microglia sampled came from control retinas whereas 34.4% of the total number of cells in the aggregate came from control retinas. Microglia from damaged retinas make up 95% of the total number of microglia captured. These findings suggest that quiescent microglia do not tolerate the dissociation and capture process for scRNA-seq as well as microglia from damaged retinas.

IL-1 signaling plays a prominent role in inflammatory responses [37]. Interleukin-1α (IL1α) and interleukin-1β (IL1β) both bind to IL-1R1 to initiate cell signaling. We queried for expression of cytokines, IL1-receptors, and associated signal transduction genes (Fig. 4f). We found that the secreted ligands *Tnfα*, *Il1β,* and *Il1α* were expressed exclusively by microglia, particularly following NMDA-treatment (Fig. 4g). *Tnfα*, *Il1β,* and *Il1α* were rapidly upregulated within microglia at 3 h after NMDA-treatment, and levels of expression remained elevated at 6 and 12 h after treatment but were reduced thereafter (Fig. 4h).*Tnfrsfr1a* is a predominant receptor for TNFα [38, 39]. *Tnfrsfr1a* expression was detected in endothelial cells, pericytes, astrocytes, and Müller glia in NMDA-damaged retinas (Fig. 4i, j). *Tnfsfr12a* was highly expressed by Müller glia at 3, 6, 12 + 24, and 36 + 48 + 72 h after NMDA-treatment and expressed in scattered microglia (Additional file 2: Figure S2). *Tnfrsfr21* was expressed at relatively high levels in scattered microglia and Müller glia in control and damaged retinas (Additional file 2: Figure S2). *Tnfrsfr1b*, *Tnfrsfr11a,* and *Tnfrsfr13b* were expressed at relatively high levels in scattered microglia, but not other types of retinal cells (Additional file 2: Figure S2). Other isoforms of the Tnf-receptor (*Tnfrsfr4, 8, 9, 10a, 11b, 13c, 17, 18, 19, 22, 23, 25, 26*) were not expressed at appreciable levels in retinal cells (not shown). By contrast, a recent study implied that retinal ganglion cells express receptors for TNFα, which is inconsistent with our findings [40]. By comparison, the receptor *Il1r1* was predominantly expressed by astrocytes, and scattered expression was observed in endothelial cells, OFF bipolar cells, and some Müller glia (Fig. 4k, l). We failed to detect *Il1r1* in microglia (Fig. 4k, l), suggesting that the reactivity and proliferation of microglia that resulted from exogenous IL1β was an indirect effect. We detected low levels of *Il1r2* in only 5/655 microglia in the retina, consistent with RNA-seq data of purified cell types from the mouse CNS wherein *Il1r2* was detected at very low levels in microglia/macrophage [10, 41]. Additionally, scRNA-seq from primate retina failed to detect appreciable levels of *Il1r2* [42]. Myd88 serves as the canonical signaling adaptor for the IL-1 pathway and functions by linking IL-1R or TLRs to kinases (IRAK 1,2, and 4) [43, 44]. Activation of IRAKs can lead to downstream activation of the NFκB pathway [45]. These IL-1R1-associated signal-transduction genes were expressed in both microglia and Müller glia from NMDA-damaged retinas (Fig. 4f). The signal-transduction components are likely involved in TLR-signaling. Thus, we probed for changes in TLR1-13 after damage. We found scattered expression of Tlr2 and Tlr3 in Müller glia and scattered expression of Tlr1, Tlr2, Tlr3, Tlr4, Tlr7, and Tlr13 in microglia (Additional file 3: Figure S3). These findings are consistent with the hypothesis that microglia do not directly respond to Il1β.

**Fig. 5:**
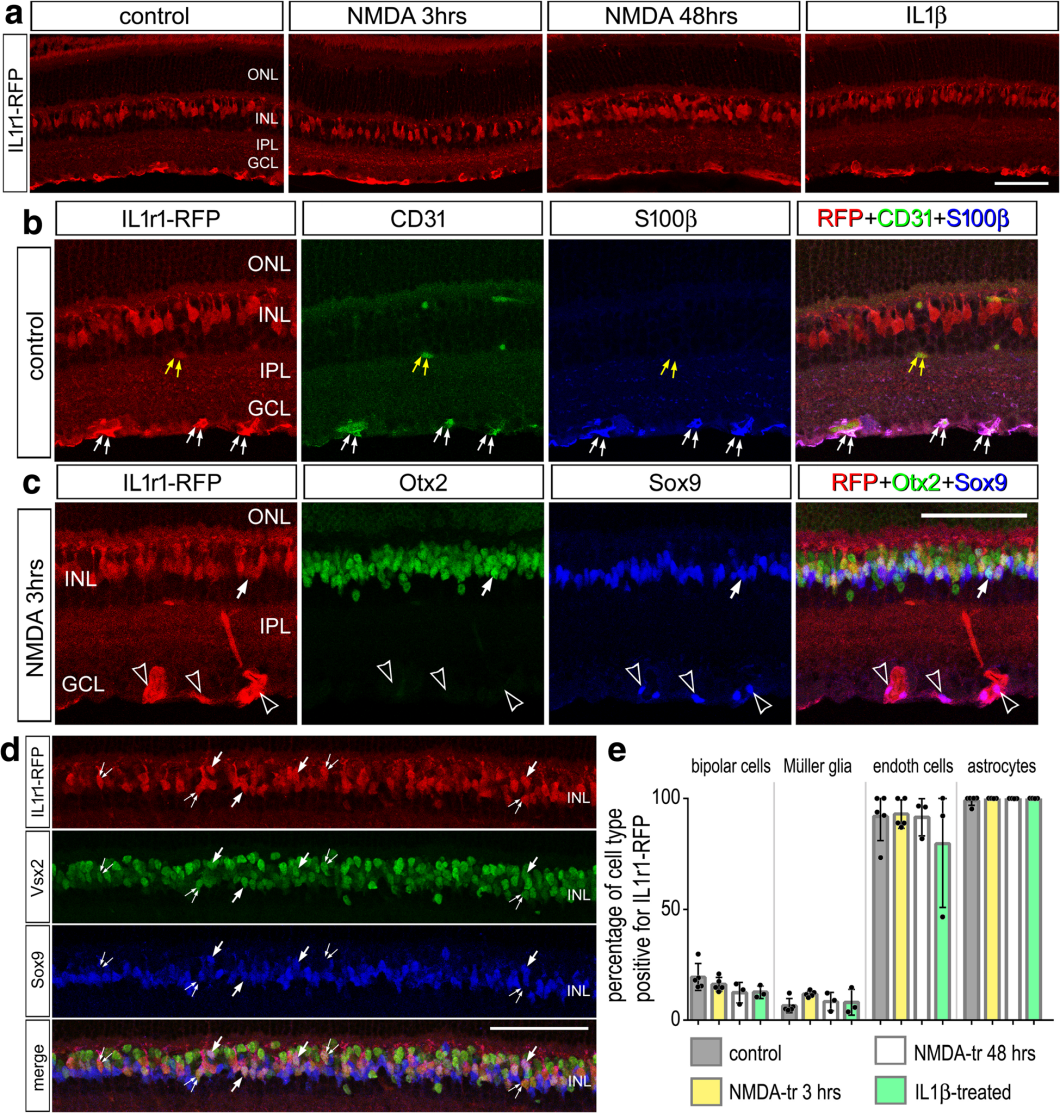
IL-1R1 expression in the IL-1R1-3HA-IRES-tdTomato knock-in line of mice. Vertical sections of the retina were labeled with antibodies to RFP (red; **a**-**d**), CD31 (green; **b**), S100β (blue; **b**), Otx2 (green; **c**), Sox9 (blue; **c** and **d**), and Vsx2 (green; **d**). Retinas were obtained from control, saline-treated eyes (**a**, **b,** and **e**), and eyes injected with NMDA (**c**, **d,** and **e**) or IL1β (**e**). Retinas were harvested at 3 h (**c**) and 48 h (**d**) after NMDA-treatment. The histogram in **e** illustrates the mean, standard deviation, and individual data points for each experimental condition. Significance of difference (*p* < 0.05) across the treatment groups for each cell type was determined by one-way ANOVA followed by Tukey’s test. Yellow arrows in **b** indicate RFP^+^ endothelial cells. White arrows in **b** indicate RFP^+^ astrocytes. Solid arrows in **c** indicate RFP^+^ bipolar cells. Open arrow heads in **c** indicate RFP^+^ astrocytes. Double-arrows in **d** indicate RFP^+^ bipolar cells, and single bold arrows in **d** indicate RFP^+^ Müller glia. The calibration bars panels **a**, **c** and **d** represent 50 μm. Abbreviations: ONL, outer nuclear layer; INL, inner nuclear layer; IPL, inner plexiform layer; GCL, ganglion cell layer; RFP, red fluorescent protein

To corroborate the data from scRNA-seq, we probed for the expression of IL-1R1 by using an IL-1R1-3HA-IRES-tdTomato knock-in mouse line in which an IRES-tdTomato sequence is inserted in the 3′ end of *Il-1r1* mRNA [20]. Consistent with our scRNA-seq data, immunohistochemistry labeling of RFP, which detects tdTomato, appeared in bipolar cells, astrocytes, endothelial cells, and a few Müller glia (Fig. 5a). Patterns of fluorescence did not appear different between control, NMDA-treated, and IL1β-treated retinas, and the expression of IL-1R1 did not vary significantly among individual cell types (Fig. 5a–e). IL-1R1-RFP was observed in nearly all endothelial cells that were CD31-positive and nearly all astrocytes that were Sox9- or S100β-positive (Fig. 5b, c, e). IL-1R1-RFP was detected in 4–10% of Müller glia that were positive for Sox9, which specifically labels Müller glia in the INL (Fig. 5c–e). IL-1R1-RFP-positive bipolar cells were positive for Vsx2 and Otx2 (Fig. 5c–e); these cells were presumptive OFF bipolar cells, consistent with the scRNA-seq data. Between 10 and 20% of the bipolar cells and 4 and 10% of the Müller glia were positive for RFP regardless of treatment (Fig. 5e). We validated patterns of RFP expression, identifying *Il-1r1* mRNA expression, by labeling for HA, which is tagged to the carboxy terminal of IL-1R1. HA-labeling is consistent with the patterns of expression seen with RFP with labeling predominant in astrocytes (Additional file 4: Figure S4).

### Exogenous IL1β activates microglia and conveys neuroprotection

**Fig. 6:**
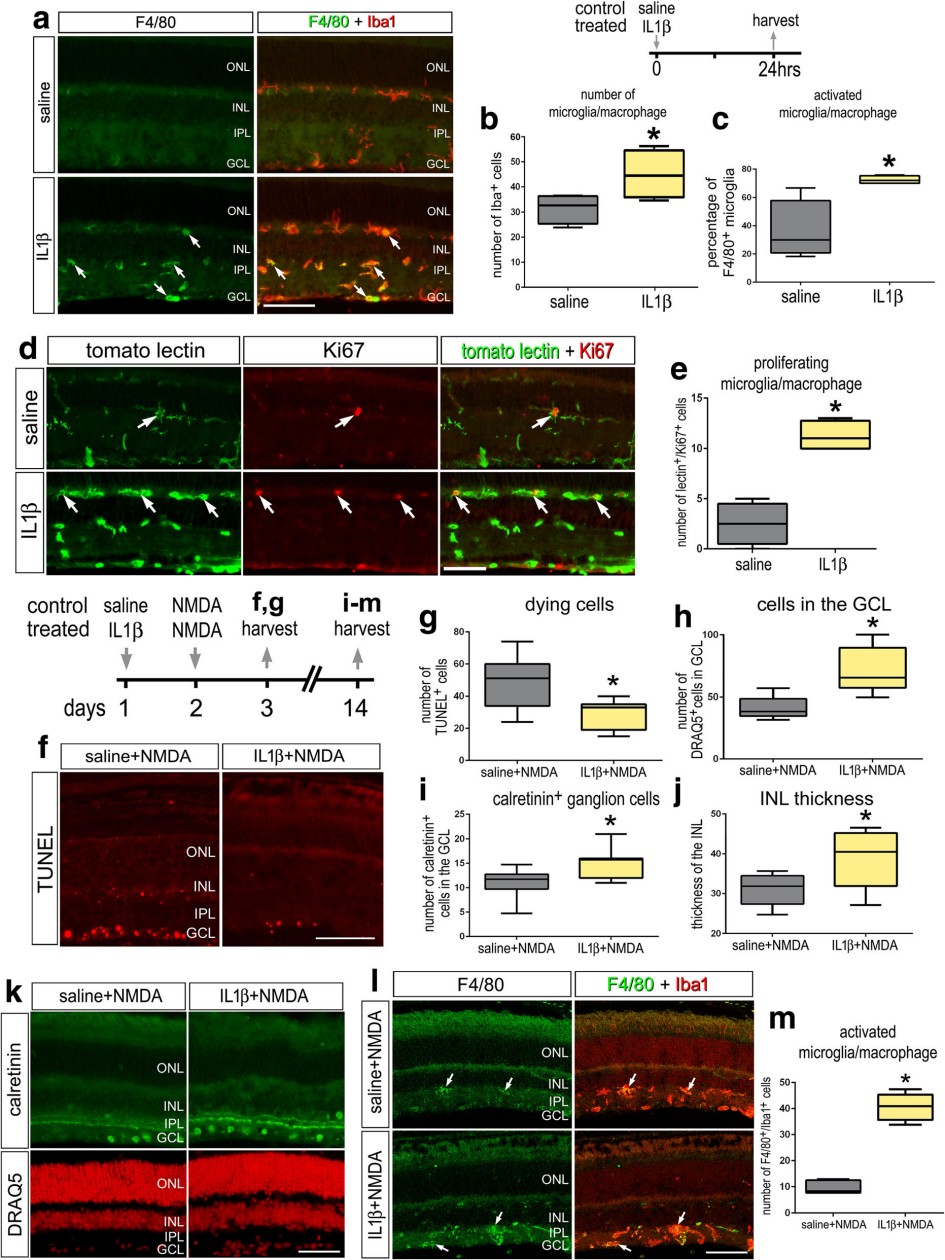
IL1β stimulates the reactivity and proliferation of microglia in the retina and conveys neuroprotection following NMDA-treatment. Eyes were injected with saline or IL1β, and retinas harvested 24 h later (**a**–**e**). Eyes were injected with saline or IL1β on day 1, NMDA on day 2, and retinas harvested 1 or 12 days later (**f**-**m**). Retinal sections were labeled with TUNEL (**f**) or DRAQ5 (red; **k**) or antibodies to F4/80 (green) and Iba1 (red; **a**), tomato lectin (green; **d**) and Ki67 (red; **d**), calretinin (green; **k**), or F4/80 (green) and Iba1 (red; **l**). The box plots in **b**, **c**, **e, g–j,** and **m** illustrate the mean, upper extreme, lower extreme, upper quartile, and lower quartile (*n* ≥ 6 animals). Significance of difference (*p < 0.05) was determined by using a *t* test. Arrows indicate microglia. The calibration bars in panels **a, d, f, k,** and **l** represents 50 μm. Abbreviations: ONL, outer nuclear layer; INL, inner nuclear layer; IPL, inner plexiform layer; GCL, ganglion cell layer

We next sought to examine the effect of the pro-inflammatory cytokine IL1β on retinal microglia. Compared to the microglia observed in saline-treated retinas, IL1β-treatment resulted in a significant increase in both the total number of microglia and F4/80-positive cells (Fig. 6a–c). IL1β stimulated the proliferation of microglia; we found a significant increase in the number of Ki67/tomato-lectin-positive cells in treated retinas compared to numbers seen in control retinas (Fig. 6d, e). There were no TUNEL-positive cells detected in IL1β-treated retinas, suggesting that cell death was not induced. These results suggest that a single intravitreal injection of IL1β is sufficient to stimulate microglial reactivity in the absence of damage, and this effect is likely to be indirect because we failed to detect significant expression of IL-1R1 in microglia via scRNA-seq or IL-1R1-reporter (Fig. 4k, l and 5).

Reactive microglia can influence the survival of neurons in the CNS, including the retina (reviewed by [4, 46–48]). Thus, we investigated whether IL1β-mediated activation of microglia influenced the survival of neurons in NMDA-damaged retinas. We found that a single intraocular injection of IL1β prior to NMDA-treatment resulted in a significant decrease in the number of TUNEL-positive cells at 1 day after damage (Fig. 6f, g). Consistent with these findings, we found significant increases in the number of Draq5-labeled nuclei in the GCL, calretinin-positive ganglion cells in the GCL, and thickness of the inner nuclear layer (INL) when assayed 12 days after damage (Fig. 6h–k). In addition, we observed increased numbers of reactive microglia labeled for Iba1 and F4/80 in IL1β-treated retinas (Fig. 6l, m). Collectively, these findings suggest that intravitreal delivery of IL1β stimulates the activation of microglia and protects inner retinal neurons and ganglion cells against excitotoxic damage.

### IL1β protects neurons from excitotoxic damage independent of microglia

**Fig. 7:**
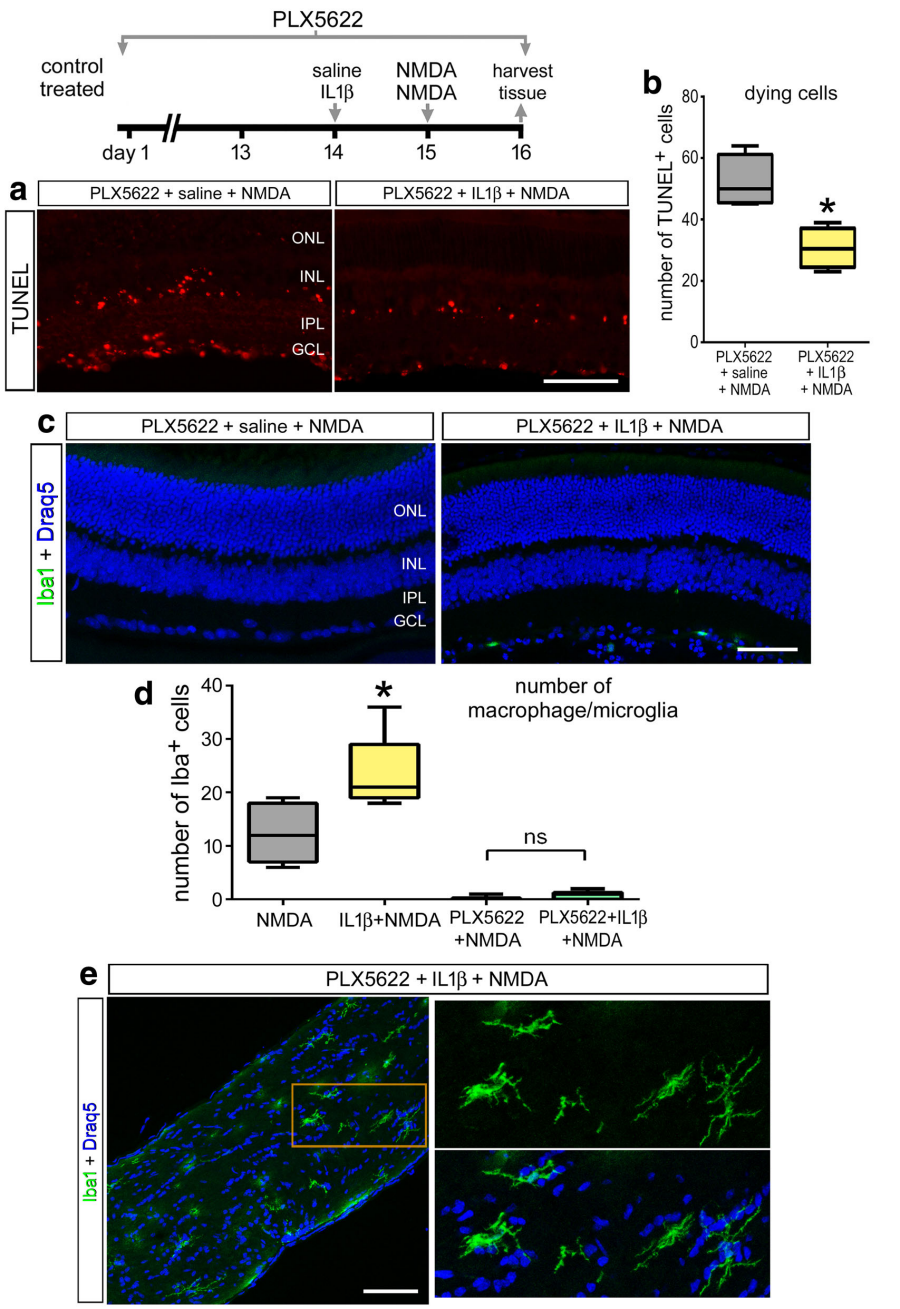
IL1β decreases cell death following NMDA-induced damage in PLX5622-treated retinas. Retinas were obtained from mice that received a diet containing PLX5622 for 2 weeks prior to a single intraocular injection of saline or IL1β at day 14 followed by a single injection of NMDA at day 15, and tissues harvested at day 16 (**a–e**). Sections of the retina were labeled using TUNEL (**a**) or antibodies to Iba1 (green; **c, e**). The box-plots in **b** and **d** illustrate the mean, upper extreme, lower extreme, upper quartile, and lower quartile (*n* ≥ 6 animals). Images were obtained from central regions of the retina (**a, c**) and within the optic nerve (**e**).Significance of difference (*p < 0.05) between treatment groups was determined using a *t* test. The calibration bars panels **a, c,** and **e** represent 50 μm. Abbreviations: ONL, outer nuclear layer; INL, inner nuclear layer; IPL, inner plexiform layer; GCL, ganglion cell layer

We next sought to test whether the neuroprotective effects of IL1β were mediated through microglia/macrophage in the retina. We found that IL1β application was protective against cell death at 24 h post injury even with depletion of microglia (Fig. 7a, b). Although we found increased numbers of microglia/macrophage in NMDA-damaged retinas that were pre-treated with IL1β compared to damaged retinas alone (Fig. 6), there was a near complete depletion of microglia in IL1β/NMDA-treated retinas from mice fed a diet containing PLX5622 (Fig. 7c, d). All regions of retinas from mice treated with PLX5622, IL1β, and NMDA were depleted of Iba1-positive cells (Fig. 7c, d). However, Iba1-positive cells were observed within the optic nerve near the optic nerve head of PLX5622-treated mice following damage (Fig. 7e). Iba1-positive cells were present after NMDA damage with or without IL1β pre-treatment (not shown). These findings suggest that IL1β treatment conveys neuroprotection against excitotoxic damage independent of the presence of microglia, and the microglia/monocytes within the optic nerve survive exposure to PLX5622.

### IL1β conveys neuroprotection by acting through IL-1R1 expressed by astrocytes

**Fig. 8:**
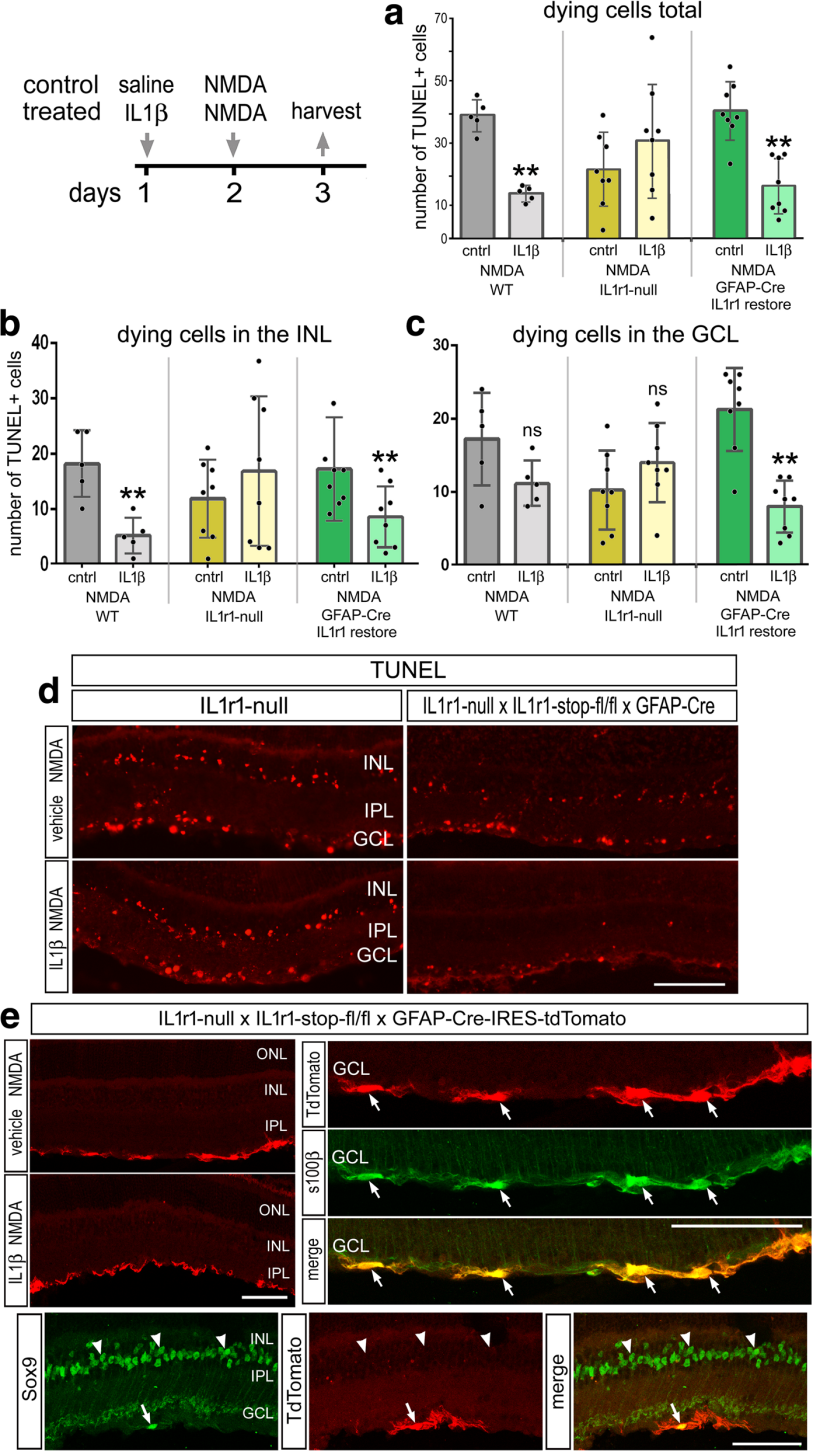
Cell death in IL-1R1-null and GFAPCre-IL-1R1r/r. Eyes were injected with saline or IL1β on day 1, NMDA on day 2, and retinas harvested 1 day later. Experiments were performed on wild-type (WT), IL-1R1-null, andGFAPCre-IL-1R1r/r. The histograms illustrate the mean (± SD and individual data points) number of dying cells across all layers of the retina (**a**), in the INL (**b**), and in the GCL (**c**). Retinal sections were labeled using the TUNEL method (**d**) or antibodies to RFP (red) and S100β or Sox9 (green) (**e**). Significance of difference (*p* < 0.0001) among the treatment groups was determined by one-way ANOVA. Significance of difference (*p < 0.05) between treatment groups was determined using a Tukey’s multiple comparison test. The calibration bars in panels **d** and **e** represent 50 μm. Abbreviations: ONL, outer nuclear layer; INL, inner nuclear layer; IPL, inner plexiform layer; GCL, ganglion cell layer

Considered together, our data suggest that IL-1R1-signaling may originate with pro-inflammatory cytokines provided by microglia/macrophage and broadly impact retinal neurons (namely OFF bipolar cells), endothelial cells, astrocytes, and Müller glia in damaged retinas. To further explore the involvement of IL-1R1-signaling, we investigated the impact of NMDA-induced damage in mutant retinas of IL-1R1r/r mice (IL-1R1-null) and GFAPCre-IL-1R1r/r mice (IL-1R1 expressed only in astrocytes).The GFAP-Cre-IL-1R1 mice are made from GFAP-Cre x homozygous knock in stop-fl/fl-IL-1R1-3HA-IRES-tdTomato line of mice that was crossed onto the IL-1R1-null background [20, 21]. With this partial GFAP-promoter, IL-1R1 is restored only in astrocytes, not in Müller glia, according to the IRES-reporter; the tdTomato-reporter was detected exclusively in S100β-positive astrocytes (Fig. 8e). Additionally, HA-labeling revealed that IL-1R1 was specifically restored in astrocytes near the vitread surface of the retinas (Additional file 4: Figure S4). We found that levels of cell death following NMDA-treatment are more variable but not significantly different in IL-1R1-null retinas compared to wild type (WT) retinas (Fig. 8a–d). The germline-loss of IL-1R1 may result in compensatory adjustments in gene expression during development that impact the variability of cell death in retinas treated with NMDA. Interestingly, injection of IL1β prior to NMDA treatment failed to provide neuroprotection in IL-1R1-null retinas. Numbers of TUNEL-positive cells were not significantly different in NMDA-damaged IL-1R1-null retinas treated with vehicle versus treatment with IL1β prior to damage (Fig. 8a–d). However, in retinas from GFAP-Cre-IL-1R1r/r mice, levels of cell death in NMDA-damaged retinas were not significantly different from those observed in WT retinas (Fig. 8a–d). In addition, the restoration of IL-1R1expression in astrocytes was sufficient to convey IL1β-mediated neuroprotection against NMDA-induced damage. Numbers of TUNEL-positive cells were significantly reduced by treatment with IL1β before NMDA-damage in retinas where IL-1R1 was restored only in astrocytes (Fig. 8a–c). Numbers of dying cells observed inGFAPCre-IL-1R1r/r retinas treated with IL1β were not significantly different from those observed in WT retinas treated with IL1β (Fig. 8a–d). Further studies are required to determine whether IL1β/IL-1R1-mediated neuroprotection involves IL-1R1 in bipolar cells, endothelial cells, and/or Müller glia.

## Discussion

We report here that microglia can provide protection to retinal neurons following an acute excitotoxic injury. After NMDA-induced damage, microglia acquire an amoeboid morphology and upregulate F4/80, indicative of an “activated” phenotype [3, 49]. Depletion of microglia via clodronate liposomes or Csf1r-antagonist prior to an excitotoxic insult resulted in increased cell death and decreased neuronal survival. By comparison, treatment of retinas with IL1β stimulated the reactivity and proliferation of microglia. This “priming” of inflammation with IL1β resulted in decreased cell death and increased neuronal survival following an excitotoxic insult, and the protective effects of IL1β do not require the presence of microglia. These pro-inflammatory cytokines are exclusively expressed by microglia in damaged retinas. Inner retinal neurons, endothelial cells, astrocytes, and Müller glia express components of the IL1-signal transduction pathway and the IL-1R1 receptor.IL1β fails to convey neuroprotection in the IL-1R1-null retina, but neuroprotection is conveyed by IL1β if the expression of IL-1R1 is selectively restored in astrocytes. Taken together, our data support the hypothesis that reactive microglia can provide protection to neurons and this may be mediated, in part, by IL1βacting at IL-1R1 expressed by astrocytes within the retina.

Microglia become “activated” during the progression of neurodegenerative diseases, but this may not always convey negative consequences. Selective ablation of microglia in an Alzheimer’s mouse model resulted in no differences in plaque load or neural survival [50]. Ablation of microglia had no effect on the survival or outgrowth of ganglion cells axons following optic nerve crush, although the depletion of microglia affected the removal of cellular debris [51]. In other contexts, activation of microglia has been associated with the onset of pathology, and suppression of microglial activation is a common strategy to promote neuronal survival and slow disease progression [4, 12, 15]. However, microglia activation is not exclusively correlated with detrimental outcomes [18, 52]. Microglia have the capacity to release anti-inflammatory cytokines and neurotrophic growth factors [53]. Furthermore, microglia can coordinate activities with Müller glia to mediate neuroprotection in the retina [48, 54]. Müller glia play a critical role in the context of retinal injury and can be beneficial or harmful to neuronal survival depending on the context [55]. Müller glia can communicate with microglia via diazepam-binding inhibitor ligand (DBI) that activates the translocator protein (TSPO) receptor on microglia [33]. DBI-TSPO signaling is upregulated during injury and inflammation and provides a mechanism for Müller glia to regulate microglia-mediated inflammation [33]. In response to activated microglia, Müller glia can release growth factors such as ciliary neurotrophic factor (CNTF) and leukemia inhibitory factor (LIF), both of which have been implicated in neuroprotection [56]. The data presented here indicate that the depletion of microglia results in increased death of inner retinal neurons following NMDA-induced excitotoxicity.

Consistent with the notion that reactive microglia can provide protection against excitotoxic damage, we found that treating retinas with IL1β prior to injury leads to a decrease in cell death and an increase in long-term neuronal survival. IL1β-stimulated microglia proliferation and F4/80 upregulation, indicating that microglia were “activated” by this pro-inflammatory cytokine. IL-1 has been suggested to be a “master regulator” of neuroinflammation and is known to be produced by activated microglia as well as stimulate microglial activation [57, 58]. Inhibition of IL1β can decrease microglial reactivity, which correlates to a reduction in photoreceptor apoptosis and preservation of retinal function in a mouse model of retinitis pigmentosa [12]. Intraocular injections of tamoxifen are potently neuroprotective and may act by reducing the production of pro-inflammatory cytokines, including IL1β [59]. In contrast to these findings, we find that IL1β application is beneficial to neuronal survival after excitotoxic damage. This discrepancy could be due to the fact that NMDA-injury is an acute damage paradigm and an initial inflammatory response may be helpful to resolve the damaged environment whereas models of photoreceptor disease may represent a model of sustained injury where prolonged inflammation is detrimental. In addition to stimulating microglial reactivity, our data indicate that IL1β may act at different types of retinal cells that express IL-1R1. Interestingly, we find that IL1β treatment is sufficient to activate microglia (Fig. 6), although microglia do not express IL1R1 (Figs. 4 and 5) This is consistent with previous studies indicating the IL1R1 is not expressed by microglia [21, 60]. These studies indicate that IL1β indirectly activates microglia by acting through IL-1R1. Liu et al. [21] showed that endothelial IL-1R1 mediates IL-1-induced activation of microglia and upregulation of inflammatory mediators. Similarly, Krasnow et al. [60] showed that IL1β-treatment results in microglial activation when co-cultured with endothelial cells or astrocytes, whereas IL1β fails to stimulate microglia when cultured alone. Similarly, we find that the survival-influencing effects of IL1β are mediated by signaling through IL-1R1 expressed by astrocytes. Astrocytes have been shown to take on reactive phenotypes that can be neurotoxic or neuroprotective. IL1α, TNFα, and C1q, secreted from microglia, have been shown to induce an A1, neurotoxic astrocyte phenotype that promotes neuronal death [61]. IL1β alone or combined with TNFα or C1q favors an A2, neuroprotective astrocyte phenotype [61]. Additionally, recent work has shown that IL1β activation of IL1R1 on astrocytes specifically represses IL-1-induced inflammatory cytokine expression in microglia [21]. Consistent with this notion, significant attention has recently focused upon retinal astrocytes as promising targets to provide neuroprotection to ganglion cells in models of glaucoma [48, 62]. Herein, we may have provided the first direct evidence that a cytokine directly acting at retinal astrocytes impacts the survival of ganglion cells in damaged retina.

It remains unknown whether the signaling from microglia to Müller glia influences neuronal survival in the retina. A previous report found that pharmacological inhibition of NFκB was neuroprotective in NMDA-damaged mouse retinas, and this resulted from decreased production of TNFα by Müller glia [63]. Production of TNFα by Müller glia was probed by immunofluorescence [63]. However, these findings are not consistent with our scRNA-seq data which indicate that *Tnfα* is highly expressed by microglia, but is not expressed by Müller glia in damaged mouse retina (see Fig. 4). Similarly, RNA-seq data from sorted cells from human and mouse brain indicate that *Tnfα* is highly expressed by microglia/macrophage, but is not expressed by astrocytes, neurons, oligodendrocytes, or endothelial cells [10, 64].The influence of NFκB-signaling on neuronal survival may be context-specific and remains controversial with findings supporting both protective [65–67] and detrimental functions [63, 68]. Further studies are required to determine the sites of action of IL1α and TNFα in damaged retinas.

NMDA elicits neuronal death via excitotoxicity and over-activation of calcium-permeable ionotropic NMDA receptors [69]. Elevated glutamate can lead to excitotoxicity in retinal pathologies including glaucoma, diabetic retinopathy, and retinal ischemia (reviewed by [70]). Considering our findings that microglia provide neuroprotection following an excitotoxic insult, targeting microglia may be a useful therapeutic strategy in diseases where excitotoxicity plays a role. Most therapeutic strategies targeting microglia in the CNS are aimed at dampening the pro-inflammatory “reactive” microglial responses that occur during chronic and progressive diseases [4]. Further studies are required to determine how reactive microglia influence neuronal survival following acute or progressive retinal damage in different models of disease. Consistent with our findings, a recent paper by Okunuki et al. [16] demonstrated that depletion of retinal microglia resulted in increased death of photoreceptors following retinal detachment. However, our findings differ significantly with those of Takeda and colleagues wherein ablation of microglia resulted in improved neuronal survival in NMDA-damaged retinas [40]. The differences between our studies may have resulted from differences in doses of NMDA, levels of damage, and activation/recruitment of microglia/monocytes and their focus on Brn3^+^ retinal ganglion cells rather than surveying survival across different types of retinal neurons.

We did not observe significant repopulation of retinal microglia following depletion, likely because we did not make observations following withdrawal of PLX5622. Sources of repopulating microglia are known to include monocytes from the optic nerve [71]. Consistent with this notion, we found Iba1-positive monocytes in the optic nerve following PLX5622-treatment which was maintained through the course of our experiments. We failed to find significant numbers of repopulating monocytes within the retina through the course of our studies. Thus, it is unlikely that reactive, residual, or infiltrating monocytes impacted the diminished survival we observed in PLX5622/NMDA-treated retinas, nor influenced the survival-promoting effects of IL1β in PLX5622/NMDA-treated retinas. Evidence suggests pathogenic roles of microglia and infiltrating macrophages in mouse models of retinal degeneration [72, 73]. By comparison, a recent study has suggested that the protective actions of microglia include recruitment of peripheral monocytes to support the survival of photoreceptors following a detachment injury [16]. The different roles of these two populations of immune cells remain unclear, as microglia and infiltrating macrophages share many immunological functions and recent studies indicate similar genetic profiles between native and repopulated microglia/monocytes [71].

## Conclusions

We conclude that reactive microglia can support survival of retinal neurons following excitotoxic injury, whereas the absence of microglia can be detrimental. In the retina, microglia are the predominant source of IL1β, which can act at IL-1R1 which is expressed by astrocytes, endothelial cells, Müller glia, and some inner retinal neurons. We find that exogenous IL1β is neuroprotective against excitotoxic damage, and this effect is predominantly mediated through IL-1R1 expression by retinal astrocytes. Thus, the influence of cytokines upon retinal astrocytes is implicated as an important therapeutic target to impact the survival of retinal neurons, particularly ganglion cells, in diseased retinas.

## Additional files

Additional file 1: **Figure S1.** Expression of *Tspo* and *Dbi* in retinal cells following NMDA-treatment. scRNA-seq was used to identify patterns of expression of *Tspo* and *Dbi* in dissociated retinal cells. Each dot represents one cell. t-SNE plots for the expression of *Tspo* (a) and *Dbi* (b). Violin/scatter plots of expression of *Tspo* and *Dbi* in microglia (c) and Müller glia (d) at different times after NMDA-treatment. The expression of *Tspo* is prevalent and upregulated in microglia and Müller glia damaged retinas. The expression of *Dbi* is prevalent in Müller glia and in microglia in damaged retinas. (JPG 3620 kb)

Additional file 2: **Figure S2.** Expression of *Tnfrsf* isoforms in retinal cells following NMDA-treatment. scRNA-seq was used to identify patterns of expression of *Tnfrsf* isoforms in dissociated retinal cells. Each dot represents one cell. Violin/scatter plots of expression of *Tnfrsf* isoforms in microglia and Müller glia at different times after NMDA-treatment. (JPG 1160 kb)

Additional file 3: **Figure S3.** Expression of *Tlr* isoforms in retinal cells following NMDA-treatment. scRNA-seq was used to identify patterns of expression of *Tlr* isoforms among acutely dissociated retinal cells. Each dot represents one cell. Cells were sampled from control retinas (rep1 5300 cells and rep2 12,932 cells) and from retinas at 3 h (8518 cells), 6 h (8000 cells), 12 h (4307 cells), 24 h (4270 cells), 36 h (1618 cells), 48 h (2246 cells), and 72 h (2269 cells) after NMDA-treatment (a). tSNE plots revealed distinct clustering of different types of retinal cells and numbers of cells surveyed (in parentheses) (b). Microglia were identified based on collective expression of *Rgs1, Trem2, Ccl3, Ccl4, C1qa, C1qb,* and *C1qc* (Fig. 4d). (c) t-SNE plots for the collective expression of *Tlr1, Tlr4, Tlr7* and *Tlr13*; expression is predominantly restricted to microglia. (d, e) t-SNE plots for the expression of *Tlr2* and *Tlr3*. Violin/scatter plots of expression of *Tlr* isoforms in Müller glia at different times after NMDA-treatment (f). (JPG 3820 kb)

Additional file 4: **Figure S4.** IL-1R1-HA is localized to astrocytes near the vitread surface of the retinas. Sections of the retina were labeled for HA-immunoreactivity in both IL-1R1-3HA-IRES-tdTomato mice and GFAPCre-IL-1R1r/r mice, which also contain the IL-1R1-3HA-IRES-tdTomato sequence. Abbreviations: ONL, outer nuclear layer; INL, inner nuclear layer; IPL, inner plexiform layer; GCL, ganglion cell layer. (JPG 3120 kb)

## Abbreviations

ANOVA: Analysis of variance
CNS: Central nervous system
CNTF: Ciliary neurotrophic factor
CSF1R: Colony-stimulating factor 1 receptor
DBI: Diazepam-binding inhibitor ligand
GCL: Ganglion cell layer
GFAP: Glial fibrillary acidic protein
IL1R1: Interleukin 1 receptor type 1
IL1β: Interleukin 1 beta
INL: Inner nuclear layer
IRAKs: Interleukin-1 receptor-associated kinases
IRES: Internal ribosomal entry site
LIF: Leukemia inhibitory factor
NFκB: Nuclear factor kappa B
NMDA: *N*-methyl-d-aspartate
RFP: Red fluorescent protein
RPE: Retinal pigmented epithelium
scRNA-seq: Single-cell RNA sequencing
TNFα: Tumor necrosis factor alpha
tSNE: *t*-Distributed Stochastic Neighbor Embedding
TSPO: Translocator protein
TUNEL: Terminal deoxynucleotidyl transferase dUTP nick end labeling
WT: Wild type
