# Supplementary figures and images for: Reactive microglia and IL1β/IL-1R1-signaling mediate neuroprotection in excitotoxin-damaged mouse retina
Source: J Neuroinflammation. 2019 Jun 6;16:118. doi: 10.1186/s12974-019-1505-5 (PMC6555727; doi:10.1186/s12974-019-1505-5)

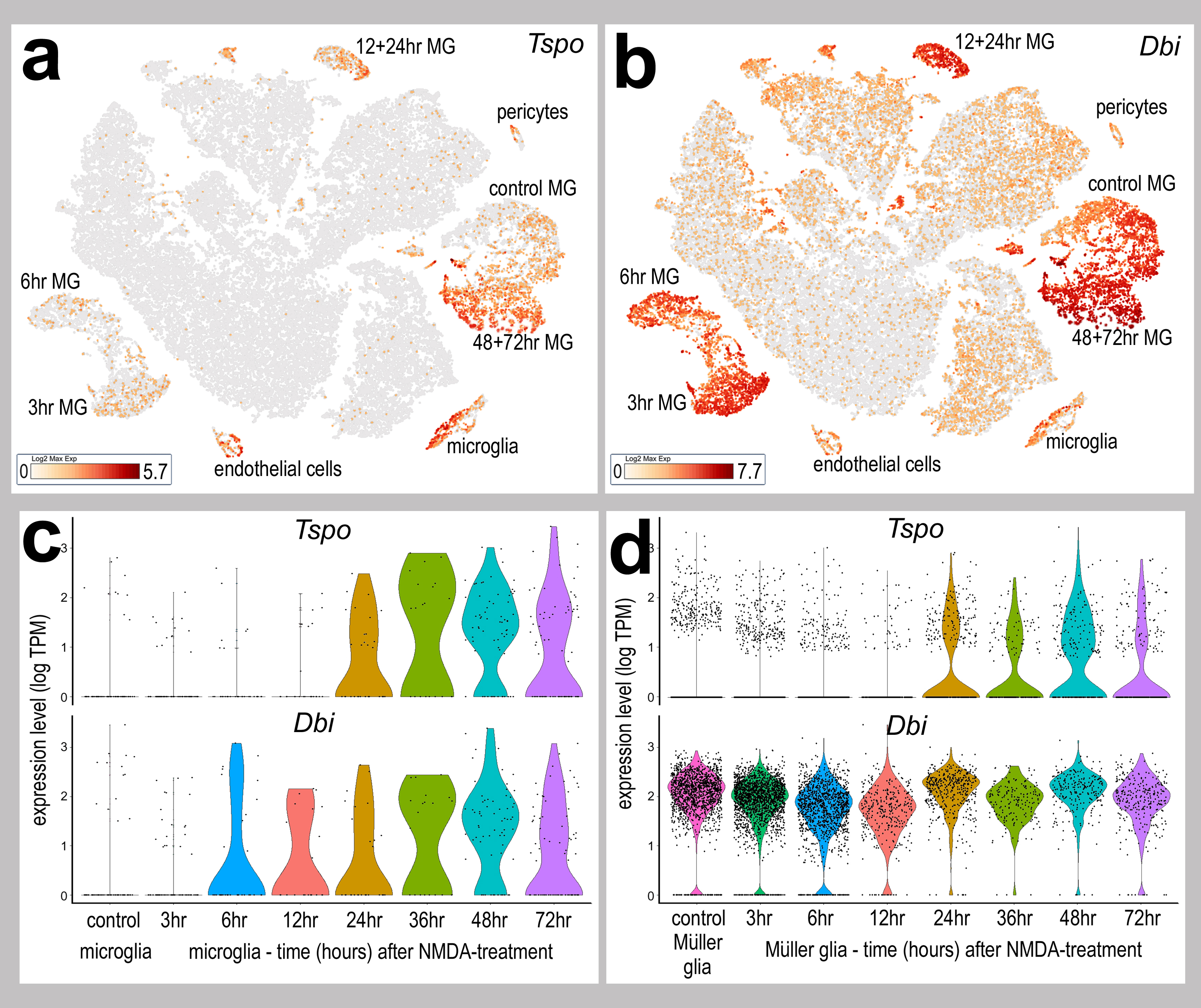

Supplement: Supplementary file 1 — Figure S1. Expression of Tspo and Dbi in retinal cells following NMDA-treatment. scRNA-seq was used to identify patterns of expression of Tspo and Dbi in dissociated retinal cells. Each dot represents one cell. t-SNE plots for the expression of Tspo (a) and Dbi (b). Violin/scatter plots of expression of Tspo and Dbi in microglia (c) and Müller glia (d) at different times after NMDA-treatment. The expression of Tspo is prevalent and upregulated in microglia and Müller glia damaged retinas. The expression of Dbi is prevalent in Müller glia and in microglia in damaged retinas. (JPG 3620 kb) [file 12974_2019_1505_MOESM1_ESM.jpg]

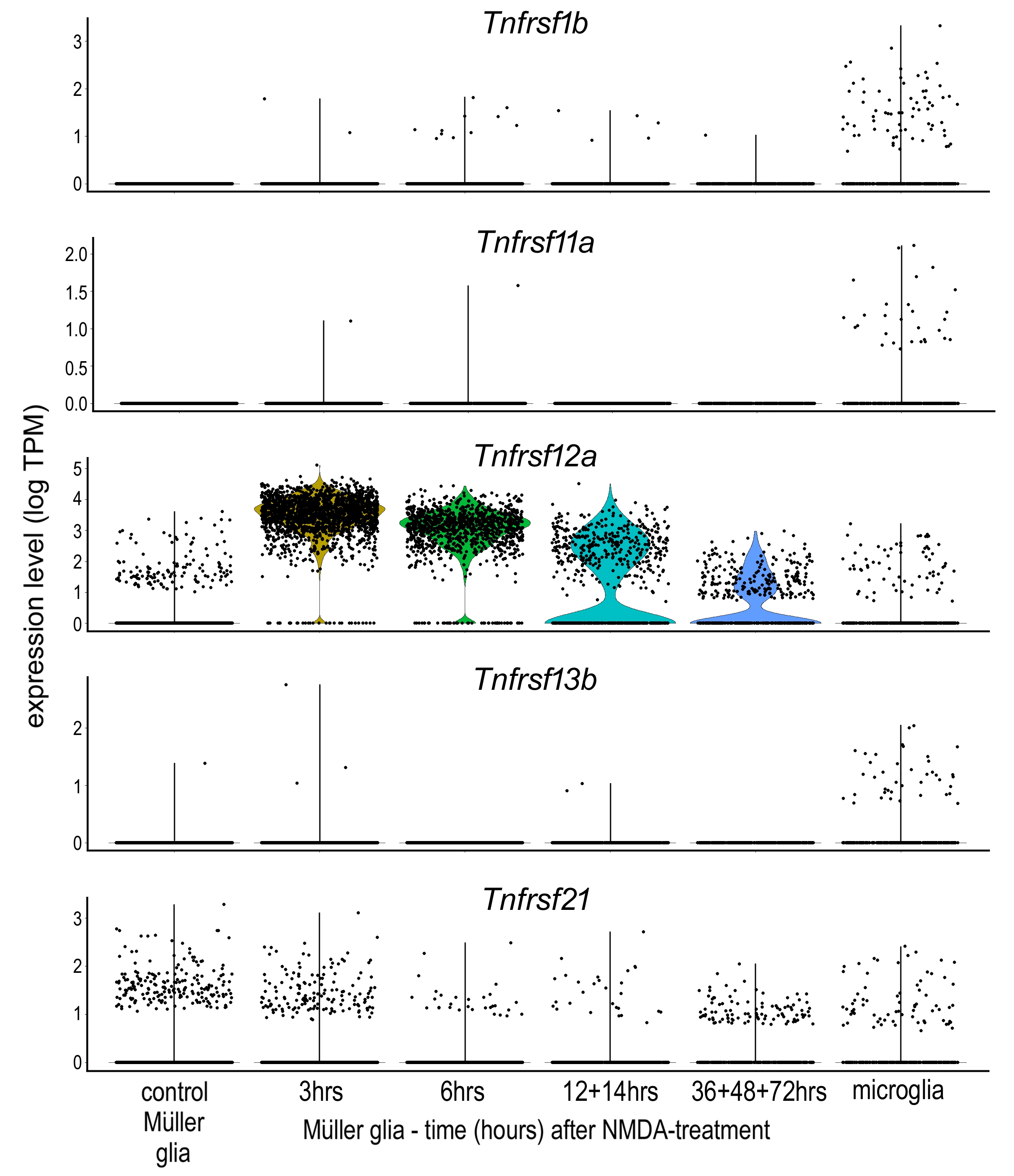

Supplement: Supplementary file 2 — Figure S2. Expression of Tnfrsf isoforms in retinal cells following NMDA-treatment. scRNA-seq was used to identify patterns of expression of Tnfrsf isoforms in dissociated retinal cells. Each dot represents one cell. Violin/scatter plots of expression of Tnfrsf isoforms in microglia and Müller glia at different times after NMDA-treatment. (JPG 1160 kb) [file 12974_2019_1505_MOESM2_ESM.jpg]

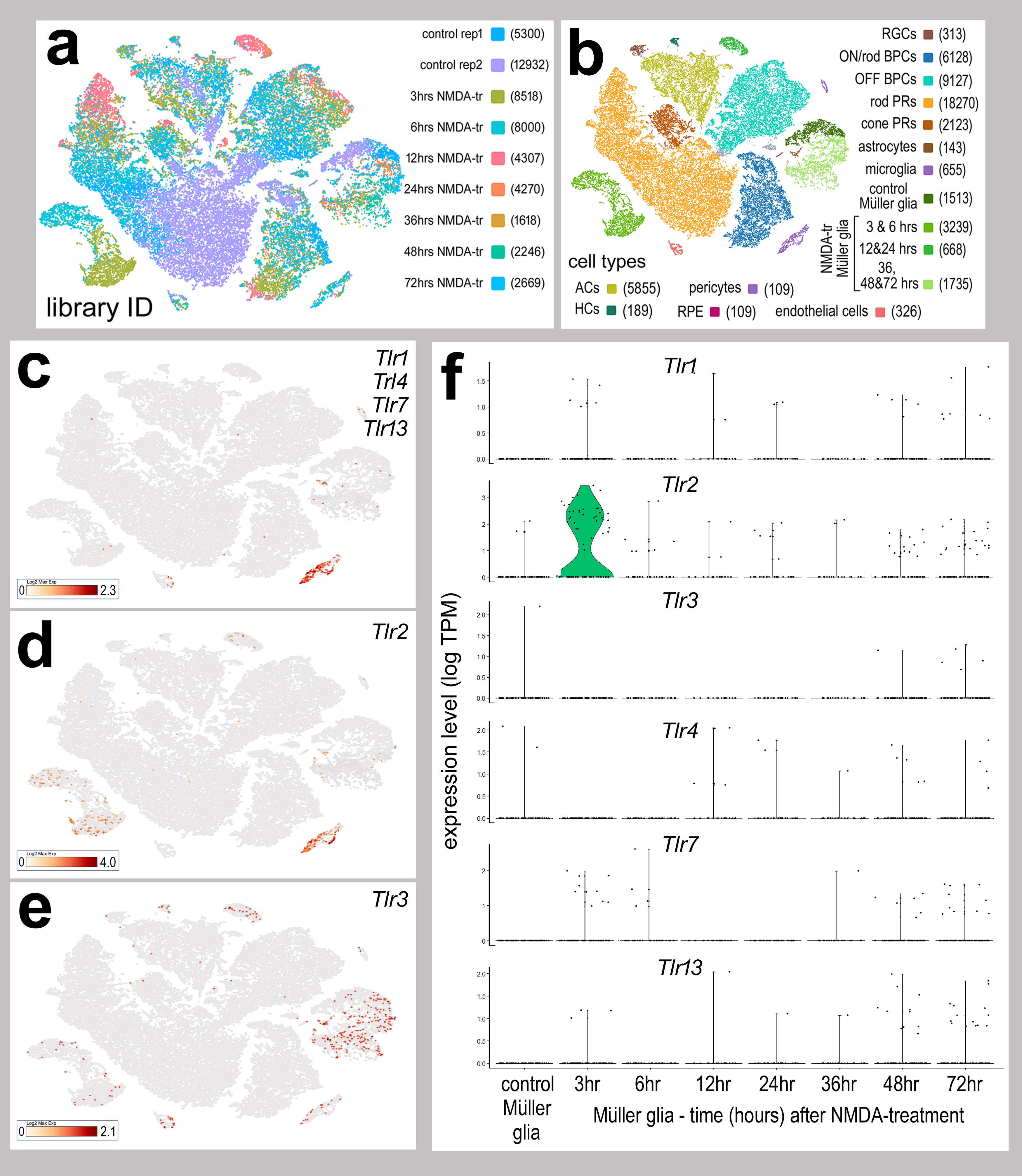

Supplement: Supplementary file 3 — Figure S3. Expression of Tlr isoforms in retinal cells following NMDA-treatment. scRNA-seq was used to identify patterns of expression of Tlr isoforms among acutely dissociated retinal cells. Each dot represents one cell. Cells were sampled from control retinas (rep1 5300 cells and rep2 12,932 cells) and from retinas at 3 h (8518 cells), 6 h (8000 cells), 12 h (4307 cells), 24 h (4270 cells), 36 h (1618 cells), 48 h (2246 cells), and 72 h (2269 cells) after NMDA-treatment (a). tSNE plots revealed distinct clustering of different types of retinal cells and numbers of cells surveyed (in parentheses) (b). Microglia were identified based on collective expression of Rgs1, Trem2, Ccl3, Ccl4, C1qa, C1qb, and C1qc (Fig. 4d). (c) t-SNE plots for the collective expression of Tlr1, Tlr4, Tlr7 and Tlr13; expression is predominantly restricted to microglia. (d, e) t-SNE plots for the expression of Tlr2 and Tlr3. Violin/scatter plots of expression of Tlr isoforms in Müller glia at different times after NMDA-treatment (f). (JPG 3820 kb) [file 12974_2019_1505_MOESM3_ESM.jpg]

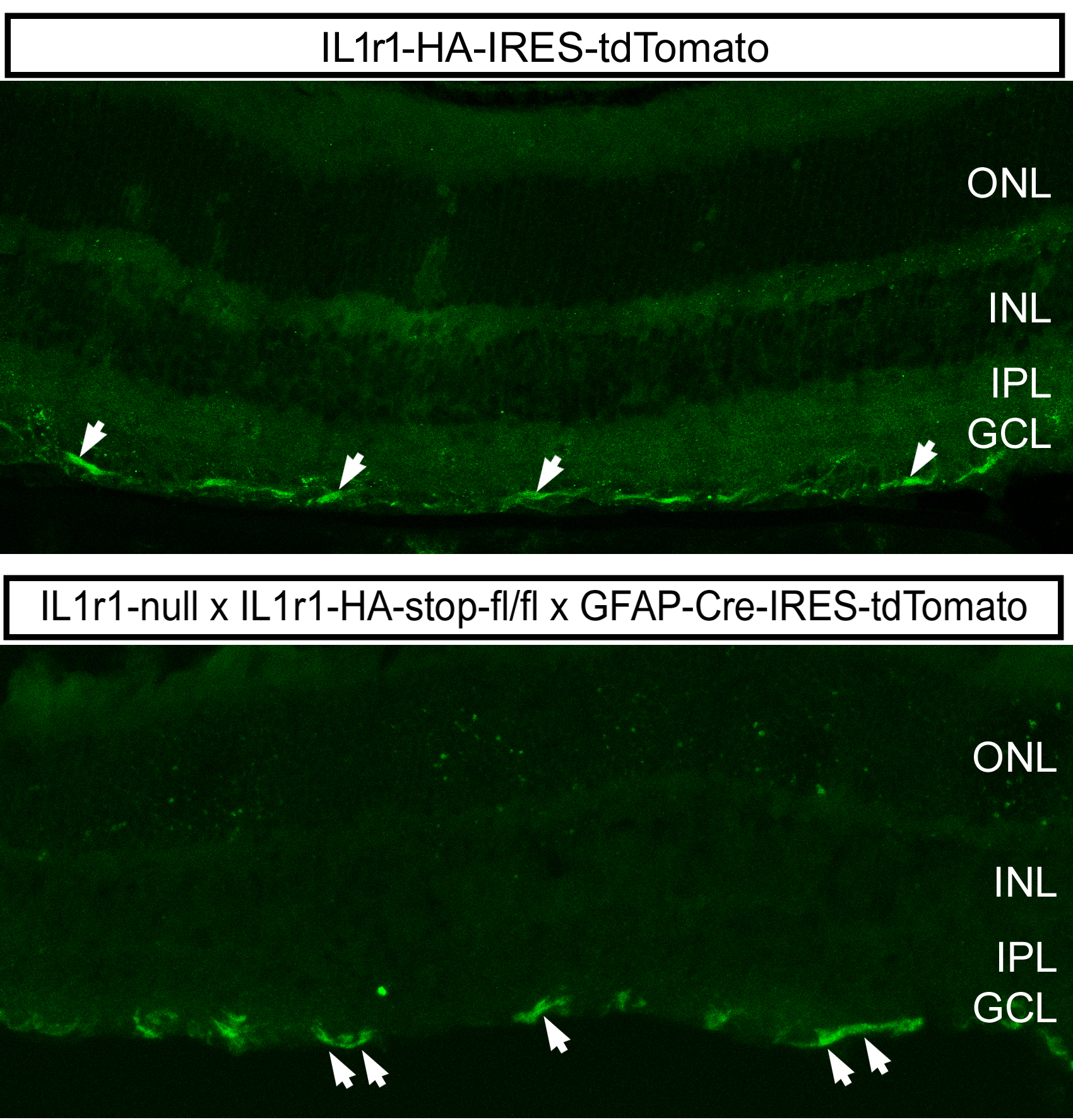

Supplement: Supplementary file 4 — Figure S4. IL-1R1-HA is localized to astrocytes near the vitread surface of the retinas. Sections of the retina were labeled for HA-immunoreactivity in both IL-1R1-3HA-IRES-tdTomato mice and GFAPCre-IL-1R1r/r mice, which also contain the IL-1R1-3HA-IRES-tdTomato sequence. Abbreviations: ONL, outer nuclear layer; INL, inner nuclear layer; IPL, inner plexiform layer; GCL, ganglion cell layer. (JPG 3120 kb) [file 12974_2019_1505_MOESM4_ESM.jpg]
